# OMGMed: Advanced System for Ocular Myasthenia Gravis Diagnosis via Eye Image Segmentation

**DOI:** 10.3390/bioengineering11060595

**Published:** 2024-06-11

**Authors:** Jianqiang Li, Chujie Zhu, Mingming Zhao, Xi Xu, Linna Zhao, Wenxiu Cheng, Suqin Liu, Jingchen Zou, Ji-Jiang Yang, Jian Yin

**Affiliations:** 1 Faculty of Information Technology, Beijing University of Technology, Beijing 100124, China2028362976@emails.bjut.edu.cn (C.Z.);; 2 Department of Neurology, Beijing Hospital, Beijing 100730, China; 3 Tsinghua National Laboratory for Information Science and Technology, Tsinghua University, Beijing 100084, China

**Keywords:** ocular myasthenia gravis, eye image segmentation, empirical study, proactive healthcare service

## Abstract

This paper presents an eye image segmentation-based computer-aided system for automatic diagnosis of ocular myasthenia gravis (OMG), called OMGMed. It provides great potential to effectively liberate the diagnostic efficiency of expert doctors (the scarce resources) and reduces the cost of healthcare treatment for diagnosed patients, making it possible to disseminate high-quality myasthenia gravis healthcare to under-developed areas. The system is composed of data pre-processing, indicator calculation, and automatic OMG scoring. Building upon this framework, an empirical study on the eye segmentation algorithm is conducted. It further optimizes the algorithm from the perspectives of “network structure” and “loss function”, and experimentally verifies the effectiveness of the hybrid loss function. The results show that the combination of “nnUNet” network structure and “Cross-Entropy + Iou + Boundary” hybrid loss function can achieve the best segmentation performance, and its MIOU on the public and private myasthenia gravis datasets reaches 82.1% and 83.7%, respectively. The research has been used in expert centers. The pilot study demonstrates that our research on eye image segmentation for OMG diagnosis is very helpful in improving the healthcare quality of expert doctors. We believe that this work can serve as an important reference for the development of a similar auxiliary diagnosis system and contribute to the healthy development of proactive healthcare services.

## 1. Introduction

Along with the development of information technology, the use of artificial intelligence to empower the healthcare industry is a major trend nowadays. In particular, the rare diseases computer-aided healthcare system may provide a solution in the areas where medical resources are scarce. An example is the diagnosis and treatment of Myasthenia Gravis (MG). According to statistics, the number of patients with myasthenia gravis is increasing year by year globally and has reached 1.1 million in 2020. It is expected to reach 1.2 million in 2030 [1] and requires lifelong testing and treatment. However, caring for MG is increasingly clustered in expert centers. On the one hand, many patients, especially in under-developed areas, can hardly get a chance to receive treatment in nearby hospitals because of the limited healthcare resources. They need to travel long distances to the expert centers, which generates a great economic burden [2]. On the other hand, it is time-consuming, laborious, and subjective for doctors to manually diagnose myasthenia gravis. Therefore, it has long been a desire to develop a convenient and cost-effective computer-aided auxiliary diagnosis system for Myasthenia Gravis, which is able to aid doctors in rapid diagnosis and help patients in under-developed areas to monitor their conditions and receive treatment suggestions from doctors remotely, so as to effectively alleviate the problem of “difficult and expensive access to healthcare”. In this paper, we present an aided diagnosis system for MG based on eye image segmentation and conduct an empirical study on the algorithm of eye segmentation for MG on this basis.

**Figure 1:**
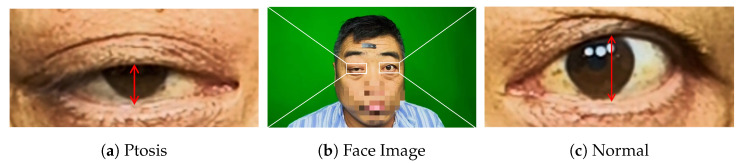
Diagram of ocular myasthenia gravis. (**a**) Right eye with ptosis. (**b**) Picture of patient’s face. (**c**) Normal left eye.

Myasthenia Gravis (MG) is an autoimmune disorder caused by autoantibodies acting against the nicotinic acetylcholine receptor on the postsynaptic membrane at the neuromuscular junction [3], and its main symptoms are weakness and fatigability of the voluntary muscles. Myasthenia gravis occurs regardless of race or gender. It usually affects women under the age of 40 and older adults between the ages of 50 and 70. But in fact, it can occur in people of all ages. Among them, the eye muscles are the main starting feature of myasthenia gravis, According to statistics, more than 50% of MG patients present with fatigue ptosis in the early stage of the disease [4], as shown in Figure 1, which we refer to as Ocular Myasthenia Gravis (OMG) [5] (i.e., only eye muscle weakness but no muscle weakness elsewhere). Without immunotherapy, 50–80% of patients will rapidly progress to Generalized MG (GMG) within two years [6], which in turn may lead to paralysis or even life-threatening, with a hospital mortality rate of 14.69% [7].

Currently, scale scoring serves as a principal method for the clinical diagnosis of OMG. Commonly utilized scales include the Quantitative Myasthenia Gravis scores (QMGs) and the Absolute and Relative score of MG (ARS-MG) among others, which aim to evaluate the severity of OMG in patients. However, the complexity of these scales combined with a shortage of well-experienced physicians, hinders early screening and cost-effective continuous monitoring of patients with OMG. This paper focuses on eye image segmentation and fully automatic OMG diagnosis. Its goal is to reduce healthcare costs in economically disadvantaged regions and enhance disease diagnosis efficiency, through which to enable precise and efficient proactive healthcare.

Studies on fundus image analysis have been made for years, laying the foundation for the development of various disease-assisted diagnostic systems specific to eye images, including diabetes mellitus [8], chronic kidney disease [9], and dry eye disease [10], Liu et al. [11] conducted an automatic diagnosis of myasthenia gravis using eye images, but they did not conduct in-depth empirical research on the eye segmentation algorithm. On this basis, we further studied the eye segmentation algorithm suitable for myasthenia gravis from two aspects of network structure and loss function, and implemented a pilot application of proactive healthcare service in practical scenarios.

Table 1 presents the examination indices for Ocular myasthenia gravis (OMG) within the scoring scale. Given the specific symptoms of ptosis or eye movement weakness in patients with this condition, we can calculate clock point (or eyelid distance) and scleral area based on the results of eye segmentation to automatically and efficiently diagnose ocular myasthenia gravis.

The motivation of the paper is to develop an eyes image segment-based automatic aided diagnosis system for OMG so that OMG patients can obtain high-quality healthcare services conveniently, remotely, and cost-effectively, and at the same time free up the diagnostic efficiency of doctors to a certain extent. Our contribution can be summarized as follows: (1) a computer-aided diagnosis system for OMG based on eye image segmentation is proposed, called OMGMed, which can effectively reduce the burden of expert doctors (a scarce resource), as well as greatly reduce the cost of patients’ access to healthcare, and make it possible to disseminate high-quality healthcare to remote areas. (2) An in-depth empirical analysis of eye image segmentation for myasthenia gravis, focusing on identifying suitable eye segmentation algorithms through the evaluation of network structures and loss functions. The empirical findings indicate that the nnUNet network structure delivers superior performance. Additionally, a hybrid loss function incorporating boundary loss enhances the eye segmentation performance for OMG, with the mean Intersection over Union (MIOU) achieving 82.1% and 83.7% in two datasets, respectively. (3) A pilot study is described for the application of the proposed system to implement proactive medical care in a real-world usage scenario. It demonstrates that our system can effectively improve the diagnostic accuracy of expert doctors. We believe that our pilot study can serve as an important reference for auxiliary diagnosis systems of myasthenia gravis and even other diseases.

The remaining parts of the paper are organized as follows. Section 2 introduces the framework of a computer-aided auxiliary diagnosis system for OMG and then analyzes the algorithmic optimization method for eye segmentation. Section 3 shows the results of the algorithmic optimization method and auxiliary diagnosis. Section 4 discusses several key issues including the results obtained and future work. Section 5 reports the case study on the application of the proposed solution to enable proactive healthcare services. Finally, conclusions are drawn in Section 6.

## 2. Methods

### 2.1. The Framework of OMGMed

**Figure 2:**
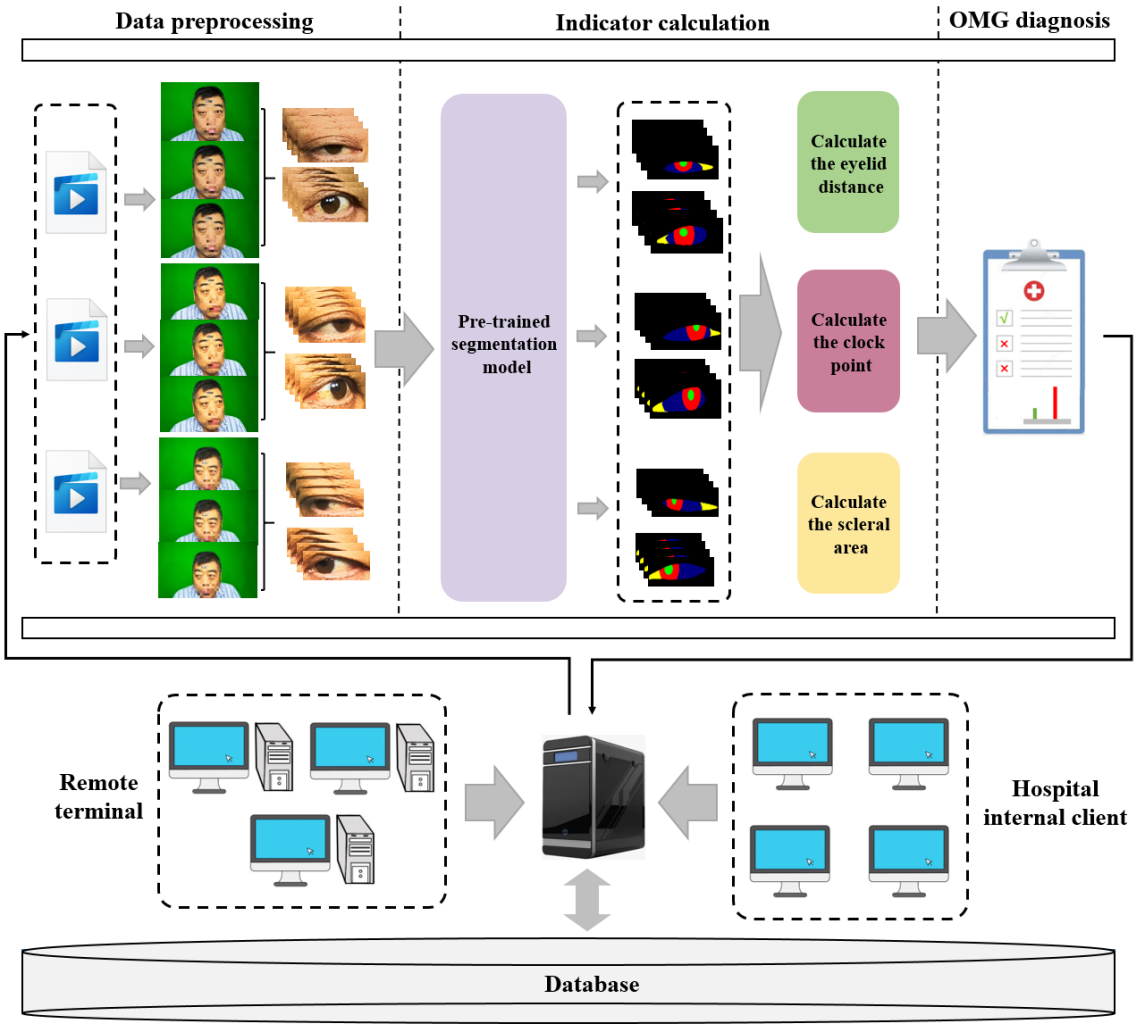
The framework of OMGMed.

The basic requirement of this research is to develop a convenient, cost-effective and efficient computer-aided auxiliary diagnosis system for OMG. Firstly, OMGMed is designed to aid doctors by providing diagnostic references for assessing the condition of patients with OMG, thereby significantly alleviating the workload of well-experienced physicians(the scarce resources) and enhancing diagnostic efficiency. Secondly, it offers patients in remote locations timely and precise insights into their conditions, minimizing the associated costs of traveling extensive distances to expert centers. As shown in Figure 2, The main component of the system consists of three parts, i.e., data preprocessing, indicator calculation, and automatic OMG diagnosis. These three parts will run on the server of a hospital and can be integrated into its existing information systems.

**Figure 3:**
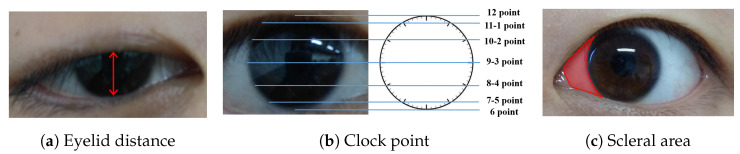
OMG indicator illustration. (**a**) Eyelid distance, distance between upper and lower eyelids. (**b**) Clock point. (**c**) Scleral area, distance between the edge of the iris and the corner of the eye.

1. **Data preprocessing module**
  Given the abundance of redundant features within facial images, we employed the face key point detection model from the Dlib library to isolate the key region of the human eye, thereby minimizing the impact of unnecessary features. Acknowledging the diverse shapes of human eyes, we extended a part of the pixels in each of the four directions to ensure the integrity of the eye in the cropped image. Finally, we get the two-eye images corresponding to the face image (left eye and right eye), and the processed two-eye images are then input into the indicator calculation module for further analysis.
2. **Indicator calculation module**
  Initially, we conducted fine-grained multi-class segmentation on the eye images. Following the segmentation outcomes, we calculated the pixel distances (area) for three key indicators: eyelid distance, clock point, and scleral area, with reference to the Common Muscle Weakness Scale: Quantitative Myasthenia Gravis Score (QMGS) and the Absolute and Relative Score of Myasthenia Gravis (ARS-MG) as depicted in Figure 3.
  **Eyelid distance:** Eyelid distance is the distance between the upper and lower eyelids when the patient is in front view and maximum eyelid view.
  **Clock point:** the cornea is regarded as a clock face, and the positions of the left and right numerical lines of the dial were used as the basis for division into seven clock positions, 12 o’clock, 11–1 o’clock, 10–2 o’clock, 9–3 o’clock, 8–4 o’clock, 7–5 o’clock, and 6 o’clock, in which the patient’s upper eyelid ptoticized to the position of the clock in the palpebral superior fatigability test.
  **Scleral area:** It is the maximum area that the sclera is exposed in the corresponding direction when the patient gazes to the left or to the right.
  After many discussions and communication with doctors in Beijing Hospital, we will no longer measure the scleral distance indicator but instead measure the scleral area. The advantages are as follows: (1) From the computer point of view, compared with a single distance, the two-dimensional area can more accurately reflect the horizontal movement of the patient’s eye. (2) From a clinical point of view, the doctor can independently use the caliper or visual estimation of distance, but can not estimate the area, therefore, measuring the scleral area can better assist the doctor in diagnosis.
3. **OMG diagnosis module**
  With reference to QMGS and ARS-MG, and after many exchanges with doctors specializing in myasthenia gravis in Beijing Hospital, we decided to use the key indicator—scleral area as the basis for diagnosis. We will calculate the proportion of the scleral area to the entire eye area, using 3% as the threshold (considering that the segmentation results may be inaccurate). If the scleral area proportion is less than 3%, we consider it to be normal; If the scleral area is greater than 3%, it indicates that the subject is unable to move the eyes normally, which is a diagnosis of ocular myasthenia gravis. From the perspective of the process, we diagnose the left eye and the right eye respectively according to the calculated indicators, and finally comprehensively output the comprehensive diagnosis results to the expert doctor or the patient.

**Figure 4:**
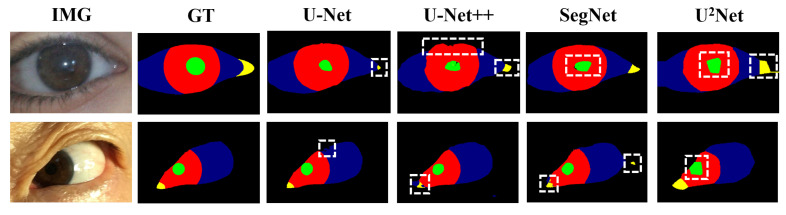
Examples of eye segmentation results where green, red, blue, yellow and black pixels represent the pupil, iris, sclera, lacrimal caruncle and skin respectively.

The framework of the diagnostic system underscores that precise segmentation of the eye is imperative for diagnosing myasthenia gravis. As the saying goes, “A miss is as good as a mile”, it becomes evident that any segmentation inaccuracies can substantially influence the indicator calculating, thereby affecting the final diagnosis’s accuracy. However, as shown in Figure 4, the performance of existing data-driven methods on multi-class segmentation of eye images leaves to be desired [12] (especially the boundary performance). The deeper rationale for the inaccurate segmentation potentially resides in the limited scale of accessible ocular medical datasets [13]. However, labeling medical datasets usually requires a high level of expertise and great labor costs [13], thus our primary emphasis centers on the resolution of this issue within the realm of deep learning methods. Intuitively, we need to take into account two factors: (1) network structure, and (2) loss function. Therefore, in the next step, we will conduct an empirical study from these two aspects and optimize the performance of the myasthenia gravis eye image segmentation model by combining the characteristics of both.

### 2.2. Analysis of Network Structure

First, we address the network structure, based on the infrastructure, we divide the existing common data-driven segmentation methods into two categories: the CNN-based network structure and the Transformer-based network structure, as shown in Table 2.

**Figure 5:**
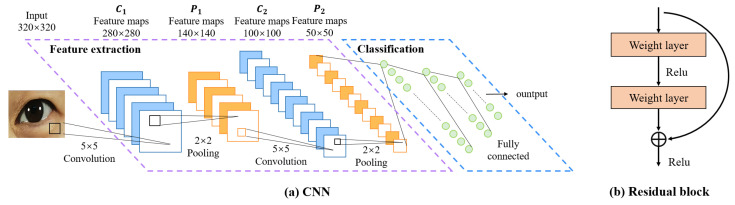
Architecture of CNNs.

**Figure 6:**
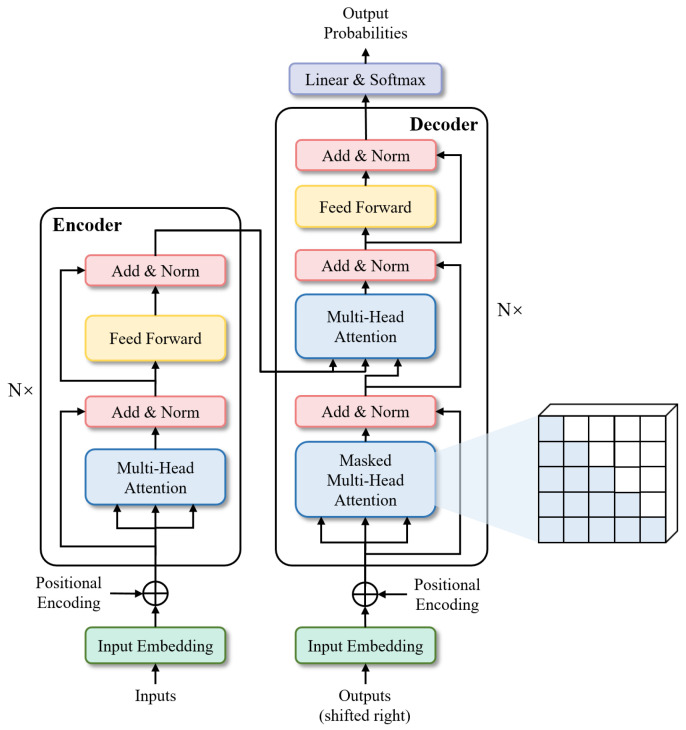
Architecture of Transformer.

1. **Convolutional Neural Networks (CNNs)**
  CNN was initially proposed by Fukushima [23] in his seminal paper on the “Neocognitron”, which is one of the most classic and widely used architectures for computer vision tasks [24]. We believe it’s also suitable for eye image segmentation. As depicted in Figure 5, a CNN is typically structured into three key layers: (i) a convolutional layer, where kernels (or filters) equipped with learnable weights are applied to extract image features; (ii) a nonlinear layer, which applies an activation function to feature maps to enable the modeling of nonlinear relationships; and (iii) a pooling layer, which reduces the feature map resolution—and consequently, the computational burden—by aggregating neighboring information (e.g., maxima, averages) through a predefined rule. In the following, we will describe the CNN structure-based network.
  - **FCN:** Fully Convolutional Networks (FCN) is a milestone in DL-based semantic image segmentation models. It includes only convolutional layers, which enables it to output a segmentation map whose size is the same as that of the input image.
  - **U-Net:** The structure of U-Net consists of a contracting path to capture context and a symmetric expanding path that enables precise localization, which was initially used for efficient segmentation of biomicroscopy images, and has since been widely used for image segmentation in other domains as well.
  - **SegNet:** The core structure of SegNet consists of an encoder network, and a corresponding decoder network followed by a pixel-wise classification layer. The innovation lies in the manner in which the decoder upsamples its lower-resolution input feature map(s). Specifically, the decoder uses pooling indices computed in the max-pooling step of the corresponding encoder to perform non-linear upsampling.
  - **U-Net++:** U-Net++ adds a series of nested, dense skip pathways to unet, with the re-designed skip pathways aimed at reducing the semantic gap between the feature maps of the encoder and decoder sub-networks
  - **Deeplabv3+:** Deeplabv3+ applies the depthwise separable convolution to both Atrous Spatial Pyramid Pooling and decoder modules, resulting in a faster and stronger encoder-decoder network.
  - **U^2^-Net:** U^2^-Net is a two-level nested U-structure that is able to capture more contextual information from different scales without significantly increasing the computational cost. It is initially used for salient object detection(SOD).
  - **nnU-Net:** nnU-Net can be considered as an adaptive version of U-Net that automatically configures itself, including preprocessing, network architecture, training, and post-processing for any new task in the biomedical field. Without manual intervention, nnU-Net surpasses most existing approaches, including highly specialized solutions on 23 public datasets used in international biomedical segmentation competitions.
2. **Transformers**
  Transformers were first proposed by [25] for machine translation and established state-of-the-arts in many NLP tasks. Illustrated in Figure 6, its inputs and outputs are one-dimensional sequences, based solely on attention mechanisms, dispensing with recurrence and convolutions entirely to enhance capabilities at modeling global contexts. This is precisely the capability required for the multi-class segmentation task of fine-grained eye images. To extend the Transformer’s application to computer vision, Dosovitskiy et al. [26] proposed Vision Transformer (ViT) model, which achieved state-of-the-art on ImageNet classification by directly applying Transformers with global self-attention to full-sized images. In the following, we will briefly describe the Transformer structure-based network.
  - **Segmenter:** Extending the visual transformer (ViT) to semantic segmentation, segmenter relies on the output embeddings corresponding to image patches and obtains class labels from these embeddings with a point-wise linear decoder or a mask transformer decoder. The network outperforms the state-of-the-art on both ADE20K and Pascal Context datasets and is competitive on Cityscapes.
  - **TransUNet:** TransUNet merits both Transformers and U-Net, not only encoding strong global context by treating the image features as sequences but also utilizing the low-level CNN features via a u-shaped hybrid architectural design. It achieves superior performances to various competing methods on different medical applications including multi-organ segmentation and cardiac segmentation.

Due to the boundary inaccuracy of the eye segmentation results (the accuracy of the boundary of the segmentation results is a key factor affecting the performance [27]). We will incorporate a boundary loss function to steer the network’s focus toward boundary pixels and explore how to hybrid the loss functions to further optimize segmentation performance in our next study.

### 2.3. Analysis of Loss Function

For the general optimization of image segmentation, current loss functions can be divided into two groups based on the scope of pixels considered by the loss function: global loss functions and local loss functions. The global loss function calculates the loss for all pixels, including both foreground (segmentation target) and background pixels; The local loss function computes the loss exclusively for foreground pixels.

- **Cross-entropy loss [28]) (CE loss):** It quantifies the disparity between the predicted value and the actual value on a per-pixel basis, considering all pixels within the image equally. It belongs to global loss.
- **Weighted cross-entropy loss [29] (WCE loss):** It further adds category weights to the cross-entropy loss, which belongs to global loss.
- **IOU loss [30]:** It only focuses on the segmentation targets, assessing the intersection and union ratio between true pixel values and their predicted probabilities. Belonging to local loss.
- **Dice loss [31]:** It only focuses on the segmentation targets, and further emphasizes the repeated computation component based on IOU loss. Belonging to local loss.

On the one hand, though the global losses empirically exhibit a high degree of stability, they tend to be less sensitive to small target segmentation, resulting in a training bias toward background classes with a larger number of pixels, and they lack a specific focus on segmentation boundaries. On the other hand, local losses concentrate exclusively on foreground pixels (segmenting the target pixels), making them less stable, and they also do not focus on the boundary pixels. To further improve the segmentation performance of OMG eye images, we introduce the Boundary loss [32].

**Figure 7:**
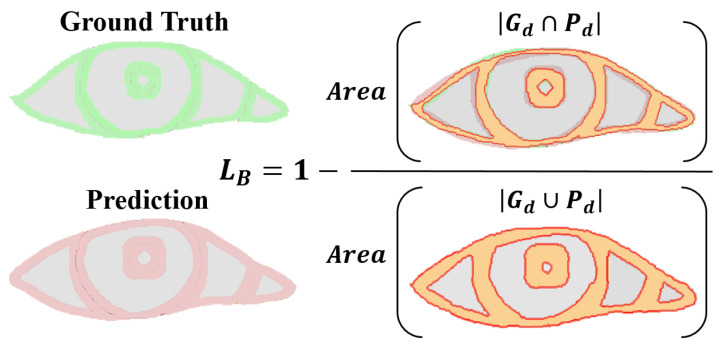
Boundary loss computation illustration. $G_{d}$ denotes Pixels from the ground truth border *d* width. $P_{d}$ denotes denotes Pixels from the prediction border *d* width.

**Boundary loss:** Focusing on the boundary pixels of the segmentation target, assessing the intersection and union ratio between true boundary pixel values and their predicted probabilities. Specifically, it only pays attention to the boundary pixels of the pupil, iris, sclera, and tear caruncle, respectively (in Figure 7), and the numerator is to multiply the predicted probability value of each category with the true value of the target pixel by pixel and then sum it; the denominator is to add the predicted probability value and the true value of each category pixel by pixel, and then to subtract the part of the “repeated computation component”. This loss has the advantages of symmetry (labels and prediction maps can be swapped without affecting computational results) and no preference (no preference for large or small targets) [33].

Table 3 provides a evaluation results of current global losses (CE loss and WCE loss), local losses (IOU loss and Dice loss), and boundary loss in terms of (1) **Stability:** Less loss oscillation during training. (2) **Boundary Concern:** Whether to focus on boundary pixels. (3) **Insensitivity:** Whether insensitive to a certain type of target segmentation. We believe that boundary loss can effectively improve the segmentation accuracy of eye images as well as the OMG diagnosis due to its focus on boundary segmentation. However, as an auxiliary loss, boundary loss usually suffers from instability since it considers too few pixels (The pixels near the boundary are often only a small fraction of the overall image pixels), thus it is more suited for supplementary roles when hybridized with other loss functions.

**Table 3:** Loss function evaluation results, where defined with the unified notation from Table 4.

	Loss Function	Definition	Stability	BC *	Insensitivity
Global loss	CE Loss	$L_{ce}=-\frac{1}{N}\sum_{i=1}^{N}\sum_{j=1}^{m}\left(y_{ij}log\left({\hat{y}}_{ij}\right)\right)$	high	×	*√*
	WCE Loss	$L_{wce}=-\frac{1}{N}\sum_{i=1}^{N}\sum_{j=1}^{m}\left(w_{i}y_{ij}log\left({\hat{y}}_{ij}\right)\right)$	high	×	*√*
Local loss	IOU Loss	$L_{I}=1-\frac{1}{m}\sum_{j=1}^{m}\frac{\sum_{i=1}^{N}y_{ij}{\hat{y}}_{ij}}{\sum_{i=1}^{N}y_{ij}+\sum_{i=1}^{N}{\hat{y}}_{ij}-\sum_{i=1}^{N}y_{ij}{\hat{y}}_{ij}}$	low	×	—
	Dice Loss	$L_{D}=1-\frac{1}{m}\sum_{j=1}^{m}\frac{2\sum_{i=1}^{N}y_{ij}{\hat{y}}_{ij}}{\sum_{i=1}^{N}y_{ij}+\sum_{i=1}^{N}{\hat{y}}_{ij}}$	low	×	—
Boundary loss	Boundary Loss	$L_{B}=1-\frac{1}{m}\sum_{j=1}^{m}\frac{\sum_{i=1}^{N_{d}}y_{ij}{\hat{y}}_{ij}}{\sum_{i=1}^{N_{d}}y_{ij}+\sum_{i=1}^{N_{d}}{\hat{y}}_{ij}-\sum_{i=1}^{N_{d}}y_{ij}{\hat{y}}_{ij}}$	very low	*√*	—

* BC means boundary concern. × and — mean the Loss does not have this attribute. *√* means the Loss has this attribute.

## 3. Experiments and Results

### 3.1. Datasets

The experiments were performed on the normal human eye dataset UBIRIS.V2 and a private dataset of eye images of myasthenia gravis patients, both of which contain multiple shots (different times, environments, and viewpoints) of the same person’s two eyes. UBIRIS.V2 is a database of visible wavelength iris images captured on-the-move and ata-distance [13], and the image acquisitions were captured using a Canon EOS 5D, containing 261 people with a total of 11,102 images, of which we labeled 3289. The private dataset is a dataset of eye images of myasthenia gravis patients that we collected from Beijing Hospital, 76 individuals, totaling 266 facial images captured with Nikon D300S (Nikon, Tokyo, Japan), Xiaomi Mi MAX2 (Xiaomi, Beijing, China), Redmi K60 (Xiaomi, Beijing, China), and Redmi Note12 Turbo (Xiaomi, Beijing, China). These images were processed to crop the eye region, resulting in 532 labeled eye images. In total, five parts of the eye are labeled: pupil, iris, sclera, lacrimal caruncle, and surrounding skin. The datasets were divided into training and test sets at a ratio of 4:1. Furthermore, to augment the dataset, the private data training set was enhanced to 850 images through image flipping.

We use Mean Intersection Over Union (MIOU), Mean Dice (MDice), Mean F1 (MF1), Mean Precision (MPrecision), Mean Recall (MRecall), Mean Boundary Intersection Over Union (MBIOU), Average time (avg_time) metrics to evaluate the model performance, where the MBIOU metric proposed by Cheng et al [33] is used to evaluate the model boundary segmentation performance. avg_time denotes the average model segmentation time.

### 3.2. Implementation Details

Our experiments are implemented by pytorch, whose hyperparameters are tuned according to the verified performance of the grid search. During training, it is trained using an SGD optimizer with a learning rate of 0.0001, the batch size defaults to 16, and due to faster convergence, the epoch of nnUnet is set to 80, while the epoch of all other networks is set to 150, and for the boundary loss, d is set to 2% of the image diagonal. All experiments were performed using a single Nvidia RTX4090 GPU(NVIDIA, Santa Clara, CA, USA). Since physiologically the pupil and iris are quasi-circular [34], we finally used ellipse fitting to fit the pupil and iris in the segmentation results as quasi-circular.

### 3.3. Empirical Experiment of Network Structure

**Figure 8:**
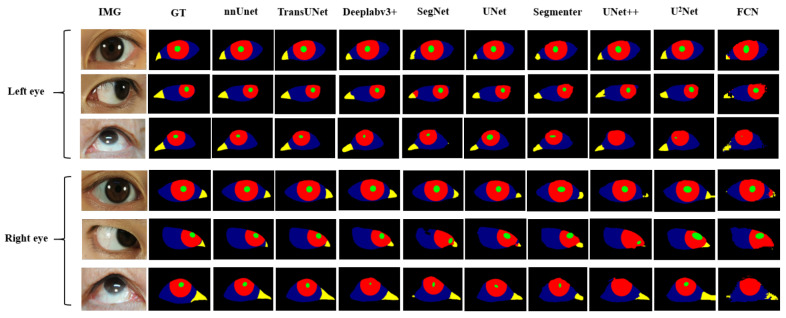
Eye images segmentation results of patients with different network.

We applied the nine networks described in Section 2.2 to segment our public and private datasets. The results, presented in Table 5 and Table 6, indicate that nnUnet, TransUNet, and Deeplabv3+ outperformed the others, ranking as the top three networks. The MIOU indexes of nnUnet, TransUNet, and Deeplabv3+ reached 81.43%, 80.89%, and 75.10%, respectively. Based on the respective characteristics of these three networks, we believe that they can well adapt to the multi-class segmentation of eye images of myasthenia gravis. As illustrated in Figure 8, which displays the segmentation outcomes from different networks. In addition, we also distinguish between the segmentation results of the left eye and the right eye. As can be seen from the Figure 8, there is no significant difference between the segmentation results of the left eye and the right eye, which indicates that the segmentation algorithm we adopted has high stability and consistency. It is not difficult to speculate that although the direction of the eyes is opposite, their external morphology and internal structure are consistent. This also proves that our strategy of data enhancement with horizontal flip is reasonable, and the consistency of both eyes can minimize the extra noise introduced after flip.

In order to optimize the segmentation performance even further, Section 3.4 we continue to explore the effect of the hybrid loss function on the multi-class segmentation of eyes. The following experiments will be based on the nnUnet, TransUNet and Deeplabv3+ networks.

### 3.4. Empirical Experiment of Hybrid Loss Function

Based on the categorization of loss functions outlined in Section 2.3, we derived three hybrid methods, namely “G + B” (G + B is an abbreviation for Global + Boundary.), “L + B” (L + B is an abbreviation for Local + Boundary.), and “G + L + B” (G + L + B is an abbreviation for Global + Local + Boundary.), and conducted experiments on both datasets. The optimal results achieved using various hybrid methods for nnUnet, TransUnet, and Deeplabv3+ are given in Table 7, Table 8, and Table 9, respectively.

**Figure 9:**
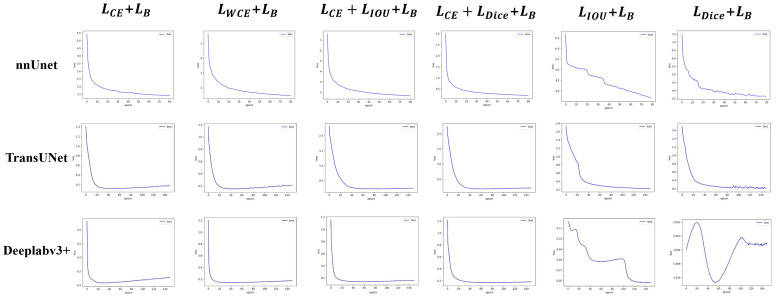
Loss of three hybrid modes on the private dataset.

The experiments validate the enhanced performance of both “G + B” and “G + L + B” hybrid modes. Specifically, In both datasets and both hybrid modes, the MBIOU of nnUnet is improved by an average of 1.0375% and the MIOU by an average of 0.5%; the MBIOU of TransUnet is improved by an average of 0.8625% and the MIOU by an average of 0.2875%; the MBIOU of Deeplabv3+ is improved by an average of 1.55% and the MIOU by an average of 0.9125%; In addition, we also conducted hybrid loss function experiments in other networks (FCN, SegNet, U-Net, U-Net++, U^2^-Net, Segmenter), and averaged the results of the above six networks, their MBIOU is improved by an average of 1.625%, and MIOU is improved by an average of 1.3%. These results underscore the robust generalization capability of the boundary loss in the context of eye image segmentation tasks. Concurrently, we observed that segmentation performance with the “L + B” model may significantly declined (an average decline of 11.625% for MBIOU and 14.52% for MIOU in two datasets and these three networks). Figure 9 shows the loss curves of different hybrid modes. Consequently, to leverage boundary loss for enhancing both overall segmentation quality and boundary precision, a global loss must first exist as the foundation. Therefore, to ensure greater stability, emphasize boundary concern, and reduce insensitivity, the hybrid loss function should adhere to the hybrid modes of “G + L + B” or “G + B”, while trying to avoid the “L + B” mode.

In order to maximize the performance of myasthenia gravis eye image segmentation, we finally decided to use the “nnUNet” network structure and the “CE + Iou + Boundary” hybrid loss function as the segmentation model of the diagnosis system by combining the network structure and the loss function aspects of the research.The specific hybrid loss function formula is shown in (1).

(1) $$L_{Hybrid}=\alpha L_{CE}+\beta L_{Dice}+\gamma L_{Boundary}$$

where α is the cross-entropy loss weight, β is the Dice loss weight, and γ is the boundary loss weight, we set α = β = 1 and γ goes from 0 to 1 with the number of epoch.

### 3.5. Accuracy of Diagnosing OMG by Doctors with the Assistance of OMGMed

We also tested the accuracy of doctors diagnosing myasthenia gravis with the help of the system. Four senior doctors, four junior doctors, and four non-doctors were invited to participate in the diagnosis, and senior doctors were defined as doctors who had worked for more than ten years or as deputy director or above; Junior doctors refer to doctors who have worked for less than ten years, and non-medical staff refers to students of other majors in the project team. We randomly selected 20 healthy people and 26 sick people as subjects, first let doctors and the OMGMed system independently diagnose, and then let doctors refer to the OMGMed system for diagnosis again. Specifically, the OMGMed system will calculate the pixel distance (area) of the three indicators of clock point, eyelid distance, and scleral area according to the subject’s eye image segmentation results, and then obtain the final diagnosis according to the relative proportion of scleral area. The segmentation results obtained by the OMGMed system, the calculation results of relevant indicators, and the final diagnosis results obtained according to the scleral area are all output to the doctor, and the doctor can refer to the above information to get the final diagnosis results. Finally, we compared the results of these three diagnoses with the real label to obtain the average sensitivity and specificity of the independent diagnosis group and the OMGMed group. Sensitivity refers to the proportion that correctly identifies the actual positives. Specificity refers to the proportion of actual negatives that are correctly identified. In addition, we also measured the approximate average time spent diagnosing a subject by various physicians in the independent and auxiliary diagnostic groups.

**Figure 10:**
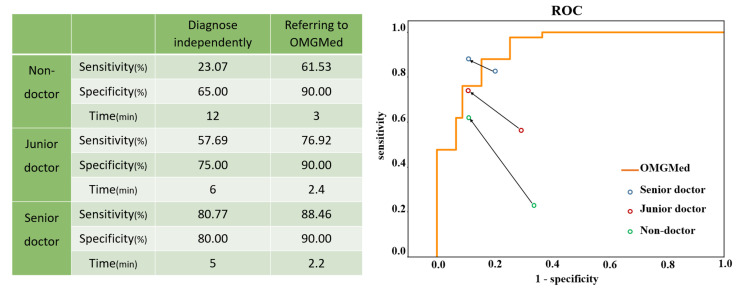
A Comparison between doctors’ independent diagnosis and doctors’ diagnosis with OMGMed. The starting points of the arrows represent the results of the independent diagnosis, and the end points represent the results with the assistance of our method. The orange line represents the receiver operating characteristic (ROC) curve of our method.

It can be seen from Figure 10 that the mean sensitivity and specificity of independent diagnosis of the junior group and non-doctor group is significantly lower than that of the OMGMed group. After referring to OMGMed-assisted diagnosis, the average sensitivity and specificity of non-medical staff were increased by 38.46% and 25%, respectively, and that of primary doctors by 19.23% and 15%, respectively. Moreover, the diagnostic accuracy of senior doctors improved when they referred to the system diagnosis, surpassing OMGMed’s automated diagnosis accuracy. In addition, the system can also improve the speed of doctors’ diagnosis, and the average diagnosis time of a subject is shortened from 7.7 min to 2.5 min. This evidence suggests that OMGMed can significantly enhance the diagnostic precision and efficiency of physicians. This system has been implemented in the MG clinic at Beijing Hospital.

## 4. Discussion

### 4.1. Eye Segmentation Performance

#### 4.1.1. Network Structure

We have carried out experiments on nine network structures in Section 3.3, covering the two major infrastructures of CNN and Transformer. In the end, the top three in terms of performance are nnUnet, TransUNet, and Deeolabv3+ in order from high to low. Based on this result, we believe that nnUnet excels in the complex multi-class segmentation of myasthenia gravis eye images, attributed to its robust adaptive capability. The encoder component of TransUNet merges the Transformer encoder’s global context modeling strength with the CNN encoder’s capacity for detailed, high-resolution spatial information extraction, which facilitates superior performance in multi-class eye image segmentation tasks for OMG. Meanwhile, Deeplabv3+ also can achieve good segmentation results due to the separable convolution and pyramid structure that expands the sensory field and captures multi-scale contextual information.

#### 4.1.2. Loss Function

The global loss has a high degree of stability, but it does not focus on the boundary pixels. The local loss has a low degree of stability, but it can focus on the whole of the segmented object. The boundary loss stability has a very low degree of stability, but it can focus on the boundary pixels. Based on the above cognition, we conducted a hybrid experiment on the three types of loss functions in Section 3.4. From the experimental results, we found that the performance of the “G + B” hybrid mode and “G + L + B” hybrid mode was improved, indicating that the boundary loss could effectively improve the eye segmentation effect. However, the “L + B” hybrid mode would lead to a great decline in performance. Combining three types of loss function characteristics, we attribute to a phenomenon of “local bias”: The model overly fixates on local outcomes to the detriment of overall segmentation results, resulting in unstable training processes and unreliable training outcomes. The loss curves of different hybrid modes also verify our conjecture (Figure 9). It can be seen that the loss function curve of the “L + B” combination oscillates greatly, indicating that the optimization direction of the model is prone to great changes during the training process due to the consideration of too few pixels. Leads to unstable and unreliable results.

### 4.2. Practical Implication

OMGMed system has improved the accuracy and efficiency of diagnosis for different groups (non-doctors, junior doctors, and senior doctors). From the perspective of accuracy, the lower the group’s professional grade, the greater the improvement of its accuracy, which is also logical. The system is most helpful to the non-doctor group, which further demonstrates the potential of the system for remote disease monitoring of patients in remote areas, illustrating the scope of the system and the wide range of applicable groups. From the perspective of time, in the past, doctors needed to use calipers close to the eyes of patients and measure the actual indicators, during which they also faced problems such as patients shaking, blinking, manual measurement inaccurate, and many times they needed to re-measure, which was very inefficient. However, the OMGMed system directly measured through eye images, greatly saving the measurement time and reducing the burden of doctors.

### 4.3. Limitations and Future Work

#### 4.3.1. Image Quality Varies

Pursuant to the results of empirical studies on eye image segmentation, we find that one dominant reason for mis-segmentation might lie in the fact that the eye images from different devices and environments have different qualities. For example, the eye image may be blurred when taken by mobile phones with low pixels; Shooting in a dark environment may cause dark areas in the lacrimal caruncle. Shooting light directly into the patient may cause the iris to appear reflected light, which is similar in color to the sclera. The above conditions are all factors that will affect image quality. Research indicates that CNNs are highly susceptible to out-of-distribution samples. It is well established that the performance of CNNs on out-of-distribution samples significantly diminishes [35].Based on the above situation, taking into account the differences in uncertain factors such as equipment, environment, and location, we plan to try to improve such problems through “data” and “model” in future work.

At the data level, Traditional data augmentation (flipping, translation, clipping, etc.) is commonly unable to extrapolate the generated data, which leads to data bias and suboptimal performance of trained models [36]. Many researchers have shown that data augmentation using GAN techniques can provide additional benefits over traditional methods [37]. Consequently, GAN techniques can be used to synthesize realistic myasthenia gravis eye images for data augmentation, thus mitigating data imbalance issues. Furthermore, for image data of poor quality, GAN techniques can be utilized for de-noising preprocessing. Cheong et al. built DeShadowGAN using manually masked artifact images and conditional GAN with perceptual loss and demonstrated the effectiveness of the model in removing shadow artifacts [38]. Although these techniques are primarily applied to fundus or OCT images, they also offer valuable insights into potential future research directions.

At the model level, firstly we can consider segmentation models built on the GAN network architecture, including conditional GANs [39], patch-based GANs [40], and topological structure-constrained GANs [41], etc. Secondly, we can focus on enhancing the robustness (generalization ability) of the segmentation model. For instance, adversarial training can be used to enhance the cross-domain generalization ability of the model [42,43,44], which introduces simulated attacks during training to bolster the model generalization ability of the crossing domains. This approach integrates segmentation consistency across different distributions of data and has great potential to be employed for improving the accuracy of eye image segmentation and automatic diagnosis of OMG.

#### 4.3.2. Single Diagnostic Basis

In the process of diagnosing ocular myasthenia gravis, our system has the problem of single diagnostic indicators. In real-world clinical diagnosis, in addition to scleral distance (area) indicators, there are many important evaluation indicators that need to be referred to, such as clock point, eyelid distance, etc. In future work, we will consider more relevant indicators as a diagnostic basis. We believe that the diagnostic accuracy of the system can be further improved after considering more indicators as the diagnostic basis.

## 5. Proactive Healthcare Service

**Figure 11:**
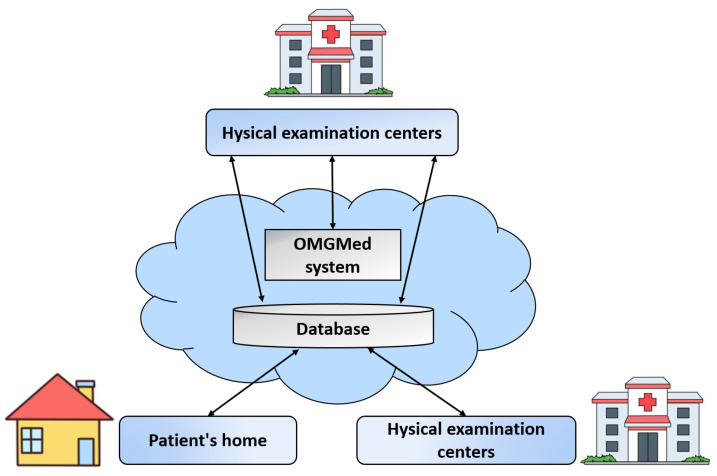
The usage scenario of the real-world application of OMGMed on eye image segmentation for automatic OMG diagnosis.

The application of OMGMed for automatic OMG detection enables the early intervention of potential OMG patients and long-term monitoring of patients with diagnosed OMG. As shown in Figure 11, the usage scenario of the pilot study on OMGMed involves three types of parties, i.e., (1) myasthenia gravis clinics; (2) physical examination centers; and (3) families of diagnosed patients. In a real-world case, parties (2) and (3) might be located in one expert center. The staff members in the party (1) are myasthenia gravis experts who provide healthcare to patients. The workers in the party (2) are nurses or general medical practitioners who work primarily on physical examinations of large populations. However, their MG knowledge is limited. The party (3) is staffed by the diagnosed patients or the patient’s families.

The operation of the OMG diagnostic solution includes two phases, i.e., the screening phase and the monitoring phase. In the screening phase, whenever the potential patients (e.g., health checkers) go to the party (2) for checking, their face data are stored remotely in the database. At the same time, the myasthenia gravis diagnostic component is triggered. Sending the original images and diagnosis results to the expert doctors in the party (1), and if the expert doctors verify the results to be true, then the party (2) contacts the potential patient and informs him/her that he/she has OMG. Similarly, in the monitoring stage, the diagnosed patients in the party (3) can upload photos at home, if the diagnosis is aggravated, the expert doctor in the party (1) will contact the patient to seek medical treatment as soon as possible, or first remote intervention.

The usage scenario in Figure 10 shows that the multiple types of objects are loosely coupled together to enable high quality healthcare to reach remote regions, greatly reducing the cost of remote patient healthcare, while also improving the efficiency and quality of diagnosis by expert doctors.

## 6. Conclusions

A computer-aided diagnosis system—OMGMed is presented, which can auxiliary diagnosis of OMG through eye image segmentation. It is helpful for improving the diagnostic efficiency of doctors and reducing the cost of MG treatment for patients, thus promoting the popularization of MG medical resources in underdeveloped areas. Building on this, we conducted an empirical study focusing on “network structure” and “loss function” to refine eye image segmentation accuracy. The experiments demonstrated the efficacy of a hybrid loss function, and finally, we selected “nnUNet” + “CE + Iou + Boundary” as the segmentation model for our auxiliary diagnosis system. The Intersection over Union (MIOU) on the two datasets are 82.1% and 83.7%, respectively.

The real-world pilot study is reported, which is about the computer-aided auxiliary diagnosis system for OMG. By retrieving eye images from the database, conducting image segmentation and automatic indicator calculation, and returning the diagnosis results to the expert doctors or patients. The study has been applied to the myasthenia gravis clinic of Beijing Hospital, which effectively improves the accuracy rate of the expert doctors. It is worthwhile to be generalized and adapted to be used for solving OMG or other medical diagnosis problems with similar situations.

## Figures and Tables

**Table 1:** Indices for ocular myasthenia gravis.

Test Item	Grade				
	0	1	2	3	4
Clock point	11∼1 point	10∼2 point	9∼3 point	8∼4 point	7∼5 point
Scleral distance	≤2 mm	3∼4 mm	5∼8 mm	9∼12 mm	>12 mm

**Table 2:** Network structure characteristics.

	Network Name	Characteristic
CNN-based structure	FCN [14]	Including only convolutional layers
	U-Net [15]	A contracting path and a symmetric expanding path
	SegNet [16]	The decoder uses pooling indices to perform non-linear upsampling
	U-Net++ [17]	Nested, dense skip pathways
	Deeplabv3+ [18]	Applying the depthwise separable convolution to both Atrous Spatial Pyramid Pooling and decoder modules
	U^2^-Net [19]	A two-level nested U-structure
	nnUnet [20]	Automatically configuring preprocessing, network architecture, training and post-processing
Transformer-based structure	Segmenter [21]	Extending the visual transformer (ViT) to semantic segmentation
	TransUNet [22]	Combining Transformer and U-Net Architecture

**Table 4:** Notation used in this paper.

Notion	Definition
*m*	Number of pixel classes
*N*	Number of pixels
$y_{ij}$	The true value of the *i*th pixel on class *j*
${\hat{y}}_{ij}$	Predicted probability of the *i*th pixel on class *j*
$w_{ij}$	Weight of class *j*
*d*	Pixel width of the boundary region

**Table 5:** Results for UBIRIS.V2 dataset.

	Network Name	MIOU (%)	MDice (%)	MPrecision (%)	MRecall (%)	MF1 (%)	MBIOU (%)	Avg_time (s)
CNN-based structure	FCN	68.40	79.27	84.96	78.03	81.35	50.39	0.0117s
	U-Net	70.16	80.44	88.38	77.16	82.39	50.50	0.0142
	SegNet	74.03	83.71	88.40	81.89	85.02	55.81	0.0136
	U-Net++	73.69	83.48	86.78	83.15	84.93	55.10	0.0186
	Deeplabv3+	75.10	84.64	83.96	88.05	85.96	56.36	0.0259
	U^2^-Net	73.77	82.54	83.16	83.89	83.52	53.37	0.0152
	nnUnet	**81.43**	**88.76**	**90.83**	**88.33**	**89.56**	**59.43**	0.0220
Transformer-based structure	Segmenter	72.47	81.65	87.47	79.13	83.09	47.13	**0.0108**
	TransUNet	80.89	88.53	90.10	**88.38**	89.23	57.72	0.0173

Bold means the data with the best performance.

**Table 6:** Results for private dataset.

	Network Name	MIOU (%)	MDice (%)	MPrecision (%)	MReca (%)	MF1 (%)	MBIOU (%)	Avg_time (s)
CNN-based structure	FCN	56.33	67.98	73.82	69.70	71.70	38.04	0.0286
	U-Net	71.24	80.87	85.64	80.22	82.84	50.64	0.0301
	SegNet	67.73	78.37	79.85	80.45	80.15	46.93	0.0327
	U-Net++	68.44	78.17	82.40	78.20	80.24	49.87	0.0332
	Deeplabv3+	72.11	81.97	80.44	87.31	83.74	49.35	0.0433
	U^2^-Net	68.49	77.73	79.36	79.25	79.30	39.81	**0.0231**
	nnUnet	**82.67**	**89.44**	**90.01**	**90.63**	**90.32**	**59.81**	0.0320
Transformer-based structure	Segmenter	71.87	80.96	85.62	79.85	82.63	44.08	0.0381
	TransUNet	81.62	88.71	89.02	89.95	89.49	57.35	0.0271

Bold means the data with the best performance.

**Table 7:** Different hybrid loss function results for both datasets of nnUnet, where the left side and right of / denote the performance on the UBIRIS.V2 dataset and that on the private dataset, respectively. α, β, and γ are the weights of losses.

Loss	MIOU (%)	MDice (%)	MPrecision (%)	MRecall (%)	MF1 (%)	MBIOU (%)	Avg_time (s)
$L_{CE}$	81.43/82.67	88.76/89.44	90.83/90.01	88.33/90.63	89.56/90.32	59.43/59.81	**0.0220**/**0.0320**
$\alpha L_{CE}$ + $\gamma L_{B}$	**81.77**/**83.40**	**89.07**/**89.96**	90.79/**90.38**	**88.89**/**91.18**	**89.83**/**90.77**	**60.76**/**61.50**	0.0291/0.0350
$L_{WCE}$	82.08/82.38	89.28/89.19	88.55/88.48	**91.75**/91.85	90.12/90.13	60.04/59.31	**0.0240**/**0.0303**
$\alpha L_{WCE}$ + $\gamma L_{B}$	**82.41**/**83.12**	**89.52**/**89.73**	**90.10**/**89.25**	90.39/**91.93**	**90.24**/**90.57**	**60.66**/**60.72**	0.0312/0.0310
$\alpha L_{CE}$ + $\beta L_{I}$	81.63/82.94	88.90/89.63	91.12/90.22	88.33/90.70	89.70/90.46	59.71/59.92	**0.0241**/0.0360
$\alpha L_{CE}$ + $\beta L_{I}$ + $\gamma L_{B}$	**82.13**/**83.64**	**89.29**/**90.03**	**91.44**/**90.34**	**88.63**/**91.29**	**90.01**/**90.81**	**60.44**/**61.75**	0.0270/**0.0320**
$\alpha L_{CE}$ + $\beta L_{D}$	82.06/83.21	89.25/89.76	91.30/90.34	**88.70**/90.91	89.98/90.63	60.45/60.90	**0.0280**/**0.0310**
$\alpha L_{CE}$ + $\beta L_{D}$ + $\gamma L_{B}$	**82.10**/**83.48**	**89.27**/**89.95**	**91.81**/**90.55**	88.54/**90.94**	**89.99**/**90.74**	60.51/**61.39**	0.0301/0.0360
$\beta L_{I}$ + $\gamma L_{B}$	69.16/70.75	73.79/74.66	75.02/74.37	73.29/75.57	74.14/74.96	51.03/50.55	0.0350/0.0320
$\beta L_{D}$ + $\gamma L_{B}$	69.16/67.71	73.80/72.43	74.95/73.23	73.35/72.85	74.14/73.04	51.03/56.96	0.0320/0.0310

Bold means the data with the best performance.

**Table 8:** Different hybrid loss function results for both datasets of TransUNet, where the left side and right of / denote the performance on the UBIRIS.V2 dataset and that on the private dataset, respectively. α, β, and γ are the weights of losses.

Loss	MIOU (%)	MDice (%)	MPrecision (%)	MRecall (%)	MF1 (%)	MBIOU (%)	Avg_time (s)
$L_{CE}$	80.91/81.62	88.53/88.71	**90.10**/89.02	88.38/89.95	89.23/89.49	57.72/57.35	0.0173/**0.0271**
$\alpha L_{CE}$ + $\gamma L_{B}$	**81.03**/**81.98**	**88.64**/**88.90**	89.41/**89.17**	**89.27**/**90.15**	**89.34**/**89.66**	**57.80**/**58.34**	**0.0161**/0.0289
$L_{WCE}$	81.11/80.67	88.72/88.19	88.41/86.63	**90.65**/**91.56**	89.52/89.02	57.43/54.94	0.0178/**0.0288**
$\alpha L_{WCE}$ + $\gamma L_{B}$	**81.19**/**81.40**	**88.79**/**88.61**	**88.86**/**87.72**	90.08/91.2	89.42/**89.47**	**58.19**/**56.87**	**0.0167**/0.0293
$\alpha L_{CE}$ + $\beta L_{I}$	80.99/81.85	88.60/88.93	89.86/88.58	88.83/**90.77**	89.34/89.66	57.40/57.92	**0.0171**/0.0284
$\alpha L_{CE}$ + $\beta L_{I}$ + $\gamma L_{B}$	**81.55**/**81.89**	**89.02**/**88.94**	**90.10**/**88.92**	**89.27**/90.61	**89.68**/**89.76**	**58.43**/**58.55**	0.0172/**0.0273**
$\alpha L_{CE}$ + $\beta L_{D}$	81.23/81.68	88.80/88.77	**90.44**/89.53	88.60/89.57	89.51/89.55	57.97/57.64	**0.0168**/**0.0283**
$\alpha L_{CE}$ + $\beta L_{D}$ + $\gamma L_{B}$	81.24/**81.74**	88.74/**88.74**	89.13/89.42	**89.84**/**89.60**	89.48/89.51	**58.29**/**58.29**	0.0169/0.0284
$\beta L_{I}$ + $\gamma L_{B}$	70.12/81.11	78.85/88.35	83.90/88.61	77.42/89.62	80.53/89.11	44.86/56.51	0.0174/0.0272
$\beta L_{D}$ + $\gamma L_{B}$	70.11/81.58	79.25/88.67	81.19/89.44	80.40/89.37	80.79/89.40	43.63/57.46	0.0174/0.0298

Bold means the data with the best performance.

**Table 9:** Different hybrid loss function results for both datasets of Deeplabv3+, where the left side and right of/denote the performance on the UBIRIS.V2 dataset and that on the private dataset, respectively. α, β, and γ are the weights of losses.

Loss	MIOU (%)	MDice (%)	MPrecision (%)	MRecall (%)	MF1 (%)	MBIOU (%)	Avg_time (s)
$L_{CE}$	75.10/72.11	84.64/81.97	**83.96**/**80.44**	88.05/87.31	85.96/83.74	56.36/49.35	**0.0259**/0.0433
$\alpha L_{CE}$ + $\gamma L_{B}$	**76.35**/**73.34**	**85.61**/**83.25**	83.76/80.22	**89.86**/**89.78**	**86.71**/**84.73**	**58.24**/**51.78**	0.0311/**0.0412**
$L_{WCE}$	75.80/73.88	85.20/83.52	82.41/79.96	90.73/90.51	86.37/84.91	57.14/51.30	0.0253/0.0425
$\alpha L_{WCE}$ + $\gamma L_{B}$	**76.84**/**75.22**	**85.95**/**84.65**	**85.71**/**81.76**	**88.33**/**90.58**	**87.00**/**85.94**	**58.67**/**54.40**	0.0253/**0.0424**
$\alpha L_{CE}$ + $\beta L_{I}$	77.20/76.14	86.24/85.08	86.66/**85.00**	**87.60**/87.86	87.12/86.40	58.97/56.06	0.0254/0.0365
$\alpha L_{CE}$ + $\beta L_{I}$ + $\gamma L_{B}$	**77.42**/**76.45**	**86.38**/**85.42**	**87.10**/84.38	87.46/**89.30**	**87.28**/**86.77**	**59.53**/**56.20**	**0.0252**/**0.0355**
$\alpha L_{CE}$ + $\beta L_{D}$	76.59/75.61	85.80/84.84	86.46/**84.62**	87.10/87.90	86.78/86.23	58.15/54.61	0.0253/**0.0428**
$\alpha L_{CE}$ + $\beta L_{D}$ + $\gamma L_{B}$	**77.29**/**76.13**	**86.29**/**85.29**	**87.20**/84.00	**87.26**/**89.23**	**87.23**/**86.53**	**59.30**/**55.71**	**0.0252**/0.0429
$\beta L_{I}$ + $\gamma L_{B}$	55.85/37.68	63.36/42.78	60.52/40.86	68.05/46.55	64.07/43.52	40.14/21.81	0.0250/0.0426
$\beta L_{D}$ + $\gamma L_{B}$	54.80/49.96	62.63/58.71	59.20/54.24	68.25/67.37	63.40/60.10	38.13/27.90	0.0251/0.0407

Bold means the data with the best performance.

## Data Availability

The data presented in this study are openly available at the following URL/DOI: http://iris.di.ubi.pt/ accessed on 20 April 2023.
